# Sources of Embodied Creativity: Interactivity and Ideation in Contact Improvisation

**DOI:** 10.3390/bs8060052

**Published:** 2018-05-23

**Authors:** Michael Kimmel, Dayana Hristova, Kerstin Kussmaul

**Affiliations:** 1 Cognitive Science Platform, University of Vienna, Liebiggasse 5, Vienna 1010, Austria; dayana.hristova@univie.ac.at; 2 Dance Studies, Creative Arts and Industries, University of Auckland, 26 Symonds Street, Auckland 1142, New Zealand; kkus802@aucklanduni.ac.nz

**Keywords:** co-creation, dance improvisation, interaction dynamics, creative mechanisms typology, self-organization, expert skills

## Abstract

Drawing on a micro-phenomenological paradigm, we discuss Contact Improvisation (CI), where dancers explore potentials of intercorporeal weight sharing, kinesthesia, touch, and momentum. Our aim is to typologically discuss creativity related skills and the rich spectrum of creative resources CI dancers use. This spectrum begins with relatively idea-driven creation and ends with interactivity-centered, fully emergent creation: (1) Ideation internal to the mind, the focus of traditional creativity research, is either restricted to semi-independent dancing or remains schematic and thus open to dynamic specification under the partner’s influence. (2) Most frequently, CI creativity occurs in tightly coupled behavior and is radically emergent. This means that interpersonal synergies emerge without anybody’s prior design or planned coordination. The creative feat is interpersonally “distributed” over cascades of cross-scaffolding. Our micro-genetic data validate notions from dynamic systems theory such as *interpersonal self-organization*, although we criticize the theory for failing to explain where precisely this leaves skilled intentionality on the individuals’ part. Our answer is that dancers produce a stream of momentary micro-intentions that say “yes, and”, or “no, but” to short-lived micro-affordances, which allows both individuals to skillfully continue, elaborate, tweak, or redirect the collective movement dynamics. Both dancers can invite emergence as part of their playful exploration, while simultaneously bringing to bear global constraints, such as dance scores, and guide the collective dynamics with a set of specialized skills we shall term *emergence management*.

## 1. Introduction: Being Creative Together

Creativity and interaction research can glean new concepts from careful qualitative research on creative events. We observed Contact Improvisation (CI) dance couples, who agreed to participate in workshop-based think-alouds. The dancers explicated in minute detail what they perceived, thought, and planned in key creative moments. This first-person viewpoint, as well as the participatory experience of two of the authors (one of them with 25 years of CI experience), enables us to contrast different sources of embodied co-creation. 

### 1.1. The Interactive Turn in Creativity and Improvisation Research

Creativity is commonly defined as behavior that combines functionality and novelty [1]. In traditional creativity theories, the subject of study are inventors, mathematicians, musicians, scientists, designers, and artists [2,3,4,5,6]. An implicit assumption dominates here that generativity is internal to the mind (i.e., individual creative achievements are in focus). 

Against this backdrop, we must appreciate critical differences in performative domains where moments evanesce and ephemerality is inscribed in the modus operandi. For example, certain postmodern dance practices are not outcome-oriented, but oriented towards a continuous process and, often, towards the esthetics of experience. They also engage as much in problem finding as in problem solving, as the creativity literature would term it. The most fundamental difference, however, is that CI and many other types of improvised dance, when practiced in duets or groups, reach beyond “internal” (i.e., mentally and individually developed) creativity. The internal represents just one pole on a wider creativity spectrum. The analysis of performative creativity demands a transactional and thus also more “externalist” view, as philosophers would say. This approach has some important precursors we can build on.

First, concessions to interactivity are made by cognitive micro-process models, which conceive of creativity as an ongoing to-and-fro between generation of ideas, exploration, selection, and idea refinement. Notably, “geneplore” theory [7,8] proposes that generation stages cyclically alternate with exploration to further interpret implications and emergent structures of ideas one has. The generation stages operate through mechanisms such as retrieval of structures from memory, formation of associations between them, as well as combination, synthesis, and transformation of these structures; they also allow for analogy building, inter-domain mappings, as well as decomposition of existing structures into components. The exploration stages, in contrast, include searching for novel attributes, implications, functions, the evaluation from different perspectives, and thinking about conceptual limitations. At both stages, resource limits and practicality concerns may constrain the process. Hence, the generation of ideas, in effect, is cyclic and evolves by recursively harnessing different resources together. In this process, *pre-inventive structures* precede the final product, which can “be generated with a particular goal in mind or simply as a vehicle for open ended-discovery. They can be complex and conceptually focused, or simple and relatively ambiguous, depending on the situation or the requirements of the task” [8]. The mechanisms for generation and exploration include mental synthesis, mental transformation, and exemplar retrieval.

The interactive range of the creativity spectrum is also prefigured by studies of design and artistic creativity which emphasize continuous explorative activity over time, such as Schön’s [9] theory of design, and in particular interactions with objects and materials [10,11]. In some kinds of creative tasks, goals and emerging means co-evolve [12,13]. In cognitive science, similar ideas have been expressed by distributed and extended cognition theorists [14,15,16] who study how “thinking” happens in interaction with objects and environments. Agents engage in continuous *solution probing* [15,17,18], where serendipity plays a role, yet explorations also have a direction and involve “intelligent fumbling” [16,19]. 

Unfortunately, however, the most highly developed creativity theories to date bypass domains that involve multiple rapidly interacting agents such as in social dances, team sports, or collaborative music making. The interest in collective co-creation has largely remained limited to phenomena such as team brainstorming, while joint *performance-based* creativity is still ill-understood. The critical difference is that co-creation, under conditions of real-time joint performance, must happen rapidly and with extant resources, yet must also be coordinated. It subjects the participating individuals to moment-by-moment adaptive pressures that arise through the interaction itself. Joint performance is in this sense inherently *improvisational*, hence a “combined behavioural and cognitive activity that requires serial creativity under tight time constraint in order to meet performance objectives” ([20] p. 350). Furthermore, since jointly improvised performances are created in real time, the question of generativity commingles with issues of rapid motor implementation and how agents precisely coordinate their activities. Consequently, the participatory dynamic over time is central for explaining co-creation, a topic that we shall briefly introduce now and pursue in depth in several later parts of this paper.

### 1.2. Theories of Performative Co-Creation

Keith Sawyer, whose seminal work covers play, improvisation theater, small-group jazz, and everyday conversation [21,22], emphasizes that joint improvisation heightens all central characteristics of group creativity [23]: First, performative domains are inherently process oriented, and different from so-called *product creativity* as we might find in invention or design. Second, there is unpredictability, with a wide range of options at any point (although this may vary from context to context), as well as combinatorial complexity. Third, meaning is determined by interaction itself; a particular action may receive its meaning only through its response by other performers (often via retrospective interpretation). Therefore, group creativity ontologically “occurs on a collaborative, social plane rather than in performer’s heads” (ibid, p. 9). Fourth, there is complex (systemically constrained) communication where the communicative negotiation between agents occurs in parallel to the actual performance. Fifth, the performance creates higher-level structures that emerge from the interactions among individuals and exhibit global system behavior.

Our present contribution will explore another of Sawyer’s central performance related notions, the idea of *distributed creativity* [22], a derivative of the term “distribution cognition” proposed by Hutchins [24]. Distributed approaches situate cognitive functions beyond the mind: in interaction dynamics, social communication channels, structured work environments, organizational infrastructures, shared procedures, and—often—tools such as charts and instruments. Concerning creativity, the key insight of the distributed approach is that interaction supplies not only the medium for joint action, but becomes its generative source. We followed Sawyer’s agenda of developing an interactional semiotics of collaborative creativity, although we aimed to do it in a more micro-phenomenologically informed way that his social theorizing would suggest and we aimed to go well beyond his sign-based semiotics. As dance creativity is mediated by embodied processes and structures, our analysis was fundamentally indebted to post-cognitivist strands of embodied, embedded, extended, and enactive (“4E”) cognition [25], with at least three tributaries to the stream.

- Interactivity theory [17,18,26] and, closely related, participatory sense-making theory [27,28], and coregulation theory [29]: These accounts de-emphasize internal mechanisms and focus on what coupling based mechanisms do for explaining collective cognition and behavior. They focus on how continuous engagements provide progressively elaborated task solutions or lead to the negotiation of new interaction frames. This school of thinking has recently spawned important enactive approaches to creativity [30,31].
- Interactional self-organization and other complexity-theory concepts have been brought into play by sports and dance scientists, who study domains such as soccer, rugby or basketball that are co-improvised as well as creative [32,33,34,35]. These authors equally de-emphasize representations and mainly model collective dynamics mathematically.
- Integrative approaches combine first and third person inquiry. Interaction-dynamic, e.g., playful, and cognitive mechanisms were combined by Brian Magerko’s group [30,36,37,38]. Some of our own work [39,40,41] also falls into this category.

### 1.3. Togetherness Constrains and Enables Creativity

Across all domains, joint improvisational ideation needs a more complex treatment than solo performances. In creative duets or groups, we see information transactions, the building of joint resources, action-based synergy, and mutual reactivity and stimulation. Experts readily agree that being creative together is more challenging than it is in soloing, because one’s actions must also fit the concurrent actions of others. Thus, togetherness constrains: Many things that can tempt a soloist are ruled out, because they would mismatch the partner’s ongoing activities (or even abilities). The necessity for finely tuned coordination and—especially in dance—for looking after one’s partner’s physical safety imposes considerable constraints. Especially when all participants have equal decision power, interaction results in strict constraints on an individual’s creativity. After all, if agents develop their own separate creative intentions, these potentially clash unless agents manage to connect or negotiate them somehow. 

On the other hand, togetherness equally confers benefits. It infuses the creative potential of individuals in multiple ways: (1) one can use immediate physical impulses between connecting bodies; (2) one can use the partner’s moves as thematic inspiration; (3) one can play, provoke, challenge, and surprise or playfully develop something together; (4) one can attain things that are physically impossible alone, i.e., “social synergies” [42] such a being lifted; (5) one can exploit re-afferences to self-produced stimuli of the partner; and (6) interactional cross-scaffolding can give rise to joint affordances or, as we show, let novel patterns emerge by and by. Thus, on the asset side, interaction itself can—if properly cultivated—become a resource and an explorative platform.

In both discussed respects, real-time coordination skills are irreducible, as studies of groups practicing jazz, classical music, flamenco, improvised theater, as well as CI [37,43,44,45,46,47,48,49,50,51,52] clearly indicate. Smooth micro-coordination furnishes the baseline for togetherness, synergy, mutual inspiration and support, the factors upon which joint creativity builds. That is, a couple who interacts smoothly is also more successful regarding creativity. Quality coordination is, thus, not mere task execution. It becomes a *principle of generation* itself, a theme we explore at length in this paper.

## 2. Contact Improvisation

CI is a dance practiced in duets, but sometimes also in trios or small groups. It is pursued with an exploratory and experiential focus [53]. We have investigated this practice using a bundle of cognitive ethnographic tools.

### 2.1. A Short Introduction to CI from an Ethnographic Angle

CI dancing thrives on playful and explorative creativity and emphasizes inter-corporeal experimentation, curiosity, and self-surprise. It plays with the tension between one’s own impulses and collaborative opportunities inspired by the moment-to-moment dynamics and inter-body configurations. The dancers are mostly engaged in tactile-kinesthetic interaction, but may move in-and-out of contact. Weight-sharing situations predominate, and when dancers are in tactile contact, momentum is used to move in concert [54]. Observing the dance, bodies appear in a rather unpredictable succession of forms. The dancers may be upright, walking, jumping, lying and rolling; they may create counter-pull configurations, build cantilevers, allow a “rolling point” to move across the partner’s body or both bodies, “slough off” (i.e., slide down) another person, go into supported handstands, flips, or lifts, and a virtually endless number of possibilities. Physical contact may be restricted to one single point of contact, or extend to a larger area of the body. Shared weight, such as leaning into each other or counterbalancing each other, opens a doorway to the creation of momentum. This invites risk-taking, reflexes, controlled falling and rising, as well as disorientation, and moving through space together. The dynamics range from high, often acrobatic speed, to slow moving with more focused investment into details. 

CI was founded in the 1970s to explore “reflex reactions of two bodies sharing weight through a moving point of physical contact” ([55] p. 42). Steve Paxton, who started the practice, describes this dance “as a spontaneous mutual investigation of the energy and inertia paths created when two people engage actively and dance freely, using their sensitivity to guide and safeguard them” ([56] p. 79). The open and transpersonal nature of the dance is what makes it interesting for its practitioners. CI dancers love to be “taken by surprise” [57]. Their joint creativity thrives on not knowing what will happen next. Dancers either look out for unfamiliar territories within familiar ones by modifying “known places” ([55] p. 42) or they move to “new places” altogether. Their dancing is motivated by “posing questions” to embodied attention [58]. Especially since CI is commonly practiced in the format of a silent jam, attentional skills are cultivated that result in a “heightened ability to sense through their skin and enhanced peripheral vision” ([55] p. 43). The inspiration to move can come from one’s own body, or from external stimuli (besides music). In exercising their creativity, dancers can choose to be completely free (apart from the physical forces that govern the movement) or follow some self-imposed task constraint or rule for some time [59].

Movement vocabulary in CI is not based on esthetic lines or shapes, but on interactively created challenges and sensation itself while playing with gravity and falling [55]. CI is known for its formal freedom and open vocabulary, especially when compared with traditional social dances. Besides safety, respect, and cooperation, CI has few constraints, being notable for its unique moments and fluidly emerging forms. All directions and body levels (upright, leaning, kneeling, lying, etc.), all kinds of interfaces (e.g., belly-to-foot, back-to-leg), and various kinds of dynamics and rhythms are admissible. Pausing or extremely subtle motion are equally possible. 

It is true that novices may at first train “ready-mades” to learn deeper principles of CI; it is also true that on a CI jam one sees a certain amount of recognizable modules such as back flips, handstands, or lifts. However, experienced dancers point out that these familiar forms can arise as reflexes of their bodies, rather than as cognitive “ready-mades”, as we explain in detail below. As the essence of CI, experts emphasize unique qualities of encounter and connection as opposed to form. For these reasons, it is also rather misguided to analyze CI through the lens of serial combinations of familiar basic elements, a way of thinking cognitive scientists have applied, for example, to Lindy Hop [60] and ballet [61]. Applied to CI, this angle would miss the point, as its dance dynamics have no set junctures or elements of a pre-determined length that would suggest such an analysis. Finally, as Torrents et al. [59] pointed out, the (experiential as well as analytic) unit of action in CI is not the individual but the biomechanical and informational macro-system formed by the two dancers.

### 2.2. Research Findings on CI Creativity

Recent studies have piloted quantitative measures of CI creativity: A study by Carlota Torrents and colleagues [34] manually codes movements in six 5-min duets and four solos into CI-typical action classes. Compared to solos, the duets tended to generate higher dynamics and variability of action, as interaction produces a “divergent productions of responses” (p. 16). Moreover, creative fluency and flexibility vary with the pairings. Another study [59] analyzes dance processes as time-series in which dancers hop between locally stable behavioral attractors, without permanently settling anywhere. The authors applied dynamic systems metrics to compare: (a) the rate/breadth of exploratory behavior; (b) the coupling strength between partners; and (c) the way in which persistent (and from a skill perspective elementary) framing dynamics such as moments with both legs and both hands on the ground provide “seeds” for shorter-lived forms. Pertinent to our present topic, these findings link the breadth of exploration and creativity with skill- and task-based constraints. First, creative breadth shrinks under extreme instructional constraints such as having to stop when someone else in a group stops [62]. Instructions to keep the pelvises close together resulted in less creativity, whereas the (more CI-atypical) instruction to keep pelvises distant apparently enforced creative solution finding (and unconstrained dancing lay mid-way). Secondly, however, task constraints vary in power at different timescales. They impact creative breadth when measured at the 10-s timescale, but at the 1-s timescale constraints from the partner seemed more essential. Finally, creative breadth, somewhat predictably, grows with skill level [59]. For example, only novices find it difficult to dance with pelvises close and therefore produce stereotypical behavior [51].

### 2.3. What Kind of Creativity Does CI Highlight?

We now turn to some basic observations about the nature of CI creativity. Dancers report that creativity benefits from sensorimotor awareness of the here-and-now and “forgetting of the past”, from serendipity and happenstances. They do not always aim at novelty for its own sake; rather a rapport-based and kinesthetically aware attitude is deemed to lead to creativity as a welcome by-product. Taking up interpersonal stimulation and the act of joint exploration are fundamental. Consequently, an esthetics encapsulated in co-action itself is valued more than “Big C” creativity, as it is sometimes called. 

Concerning the novelty aspirations of CI dancers, various loci may be distinguished: The most obvious locus lies in new forms, configurations, and dynamics, including hybrids of known material. The novelty here can consist in athletic virtuosity or ease of execution, or the relation to the own or other body and/or surrounding spatiality. Similarly, dynamic qualities of the dance can be exceptional, and some dancers like to invest much creativity, for example, into the “musicality” of the dance. The fact that dancing has a sequential aspect to it opens the possibility of building meaningful temporal gestalts, rhythms, and combinations. Aspects of such *serial creativity* can go as far as creating “narrative” developments or motif building, often at the timescale of minutes. In the smaller interstices of the moment, we observe two phenomena: On the one hand, virtually all dancers seek novelty at the interpersonal level concerning communication, as well as curiosity about partner reactions and the affective dynamics between the partners. On the other hand, dancers can fill “familiar places” with new qualities, including micro-dynamic qualities, communicative qualities, a new relationship to context, or a hitherto unachieved naturalness of initiation from ongoing dynamics. Even a handstand can be creative with respect to how it figures in context and how it arose dynamically. One dancer spoke of “a new pathway to a situation that itself was …more conventional”. Thus, new contexts, accentuations, specific new tactile or proprioceptive qualities, as well as dynamic variations may endow what otherwise would appear as a “ready-made” with creative qualities. Accordingly, our experts agree that creativity can relate to subtle, even unobservable qualities in the movement or nuances of perception. Here, the creative “how” is more in focus than the “what”. Both the communication level and the qualities of movement can be captured by what we would term *micro-creativity*. Occasionally, CI practice is also open to transformational creativity [63] which shifts the implicit boundaries and conventions of a domain (or creates genre hybrids such as Contact Tango). 

From a comparative angle, and informed by questions that speak to the general creativity literature, seven defining characteristics of CI creativity meet the eye: 

(1) As mentioned, CI is process-oriented and improvisational. A constant stream of action decisions is made without delay, using present resources, and in response to current constraints and adaptive pressures. Any creative process must build on what is readily at hand, often rapidly. Although creative processes need not be exceptional, great priority is placed on the action being highly situation-aware, and transparently communicated.

(2) CI creativity is biomechanically constrained and spatially located. Whatever comes next must fit the current situation. The dancers cannot skip between constellations in space as a keyboard player might. CI practitioners always need to pick up from where they are and their actions should offer continuation options for the moment that follows. Although this is not a strict constraint, constancy and continuous movement flow are considered important. Lesser constraints operate on the level of sequentiality. Although dancers continuously add to an emergent structure (Sawyer speaks of “emergents”, [23]), the movements are causally cumulative only to a limited degree, i.e., only when acrobatic moves demand closure for the sake of safety. (This contrasts with improvisation theater where additions have to make sense retrospectively and with respect to prospective narrative closure [21], and with soft martial arts where a defence is built up over several stages [64].)

(3) Most CI creativity happens intercorporeally, through joint kinesthesia. Joint exploration of space, mutual sensory explorations of the partner’s body, functional interpenetration with the partner are vital, where a macro-system of two bodies arises. Sharing surface contact provides the ideal medium for ceaseless negotiation. At the same time one adapts to the partner, the partner is adapting to oneself. Analytically, we may speak of mutual incorporation and kinesthetic interconnectivity [65,66,67], and a bidirectional coupling between sensorimotor systems that allows minimally delayed reactions as well as co-modulations of ongoing contributions of the partner. At many moments, shared weight systems or force vectors that run through both bodies are maintained through touch. The moment when weight is carried through another moving body is full of potential for encounter, surprise, and emergence.

(4) Even when dancers are not aiming for creativity, they are ready to react and to adapt to momentum or gravity. After all, the partner might always come up with an idea, the configuration may flip into something different, or new short-range goals emerge. In fact, dealing with surprises, novelty, and emergent risks is the dancers’ raison d’etre. Creativity acts often arise as response to biomechanical adaptation pressures. What counts as adaptive reaction can mean different things in CI: In many instances efficiency and biomechanical economy are given priority, yet in others dancers do not follow the natural path of energy flow, but seek creativity by leaving their comfort zone and by courting risk on purpose (albeit without compromising the quality or continuity of rapport).

(5) In addition to biomechanically necessary adaptations, interior experiential qualities in the ephemeral present are cherished. CI has a domain-specific esthetics that goes beyond just managing one’s way out of tricky situations or the search for physical efficiency alone. We need to think of CI as exemplifying a sort of “survival-creativity-plus” (to adapt Torrance’s [68] idea of *survival creativity*). CI embodies what creativity researcher Welling [6] has noted more generally: the implicit selection criteria that determine what an acceptable solution is comes from a mix of survival value and esthetic criteria. Although the dancers need to mind safety before getting creative, CI cultivates embodied curiosity for its own sake and, furthermore, provides an arena to explore cultural semiotics of touch and kinesthesia. In its experiential orientation and exploration, CI is similar to other art forms, and challenges merely adaptability-focused theories of creativity [69]. 

(6) CI creativity is explorative and playful. The participants regard the joy of discovery and curiosity as purposes in themselves, an embodied esthetics with immediate affective payoffs [38,70]. Time and time again, we have heard emphasized the gratification of sharing physical exploration and surprise. As in any playful activity, problems need to be found or even invited rather than being set. In this capacity, CI combines problem solving (albeit not always of well-defined problems) with problem finding [71]. Another factor related to playfulness is that the dance system is kept very malleable and responsive. The degree of constraints dancers wishes to set at a given moment is largely up to them (as opposed to social dances such as salsa or tango with a strict “grammar”).

(7) CI creativity is not only a joint process, but also a constantly interactive one that links the dancers in a double loop of simultaneous feedforward and feedback. This type of interaction has been called *coregulation* [29], a real-time process of meaning making, or—as we might say—of continuous *decision-making-in-action* [64]. The dancers source themes, inspirations, challenges, as well as solutions from the transaction of information and physical momentum. Since information flows and actions are mostly simultaneous (and in this respect quite unlike conversational turn-taking) physical impulses can mix into something genuinely new. Hereby, the creative process may become interactive in a deeper ontological sense. Collective dynamics and the *participatory* process of engagement over time [72] drive generativity (see Section 5.7 and Section 5.8).

## 3. Methodology

After this general outline, we are ready to introduce the particular methodology used in this study. As a first step in our data collection, we conducted dialogical interviews with five individual CI dancers. The participating dancers have been practicing CI for 15 years or longer, and teach CI internationally. One of our informants, Nita Little, co-developed CI together with CI founder Steve Paxton and others in the early 1970s and has been teaching it since. The interviews gave us an idea of our informants’ dance personalities and histories; as well as allowing us to probe resources, personal styles, and creativity interests. In a second step, we aimed at a genuine *interactional semiotics* for joint improvisation [23], using methods that are incident-based and can provide a high-zoom factor on the process of co-creation. 

Specifically, we organized 21 think-alouds in the dance studio with pairs of dancers in sessions that lasted between 2.5 and 4.5 hours. Our aim was to closely track unique dance incidents of approximately 2–7 seconds, which we had selected together with the dancers. To elicit micro-genetic descriptions, we then encouraged the dancers to inspect the event from different angles. Recursive probe question were asked and our informants were required to stay close to the sensorimotor level while abstaining from generalizations. A detailed description of short perception–action cascades emerged from this dialogue, complemented by a final synopsis, comparisons to similar events, and how the event related to their dance experience in general (hence, we wrapped up by allowing a certain amount of more general reflections). On a few occasions, we elicited responses during the dance, but predominantly we engaged in a dialogue immediately after dancing while the dancers inspected video feedback on a tablet computer. In several of the think-alouds, we also used quasi-experimentation. For example, a dancer was asked to strategically perturb the interaction so we could observe the partner’s adaptations. To better understand adaptive constraints, we also asked dancers to progressively degenerate a specific movement pattern or subtly alter the context until another action strategy became more attractive.

Throughout, we employed micro-genetic interviewing techniques that help informants access tacit or embodied knowledge and elevate it above the threshold of consciousness. A tried-and-true micro-phenomenological method, the Explication interview [73,74,75], was customized for topics in sensorimotor interactions. This format allows informants to jointly explore their experience in a dialogue. The researcher’s role is to help sustain attention on a precisely circumscribed micro-experience, often a decision or pre-decision moment. Explication interview techniques are known to enable informants to verbalize subtle details of their embodied coping skills, and allow for tacit knowledge to become accessible in the first place. The sustained attention allows “blowing up” micro-moments of around one second or less for fine-grained inspection. 

In the subsequent data analysis, the (recursive and cyclic) questions were reworked into a timeline model that shows how the dancers mutually cue and enable one another. Thus, detailed scores of dance snippets were reconstructed, as to who felt what when and where, which affordances were recognized, what information they consisted of, when actions set in, how actions complemented each others, or when transitions set in. 

Up to now, 20 think-alouds were transcribed and analyzed, which we screened for instances of *conspicuous creativity* (defined as psychological creativity after Margaret Boden [63], not historical creativity). We then matched up the most notable examples with theories of creativity and from this created a functional taxonomy of creativity types. Short empirical examples to illustrate this taxonomy, as well as a more detailed process vignette, are reported in Section 5. (For space reasons, we had to condense the extended case descriptions; we instead supply selected quotations, which—although incident-based—were selected to be representative of our data pool.) Moreover, our think-alouds and interviews led to a useful survey of creativity skills in CI, as we report next. 

## 4. Results I: Skills that Make Dancers Creative

Our survey of empirical findings begins with skills that our dancers deem central. In the field of CI, embodied and creative abilities cannot be neatly disentangled, since the latter presuppose the former. By degree, however, we may distinguish: (a) skills that enable joint creativity and provide conditions of possibility, as it were (Section 4.1, Section 4.2, Section 4.3 and Section 4.4); and (b) creativity techniques in the narrower sense (Section 4.5). 

### 4.1. Enabling Sensorimotor Skills

Our team has learned from various previous studies on martial artists, dancers, and bodyworkers that improvisation never happens out of the blue. It presupposes highly prepared background structures and a readiness of mind and body. We therefore began by asking our CI informants specifically which habits kick in when the dance begins and how they bestow a “joint-improvisation-friendly” structure on their bodies. Precise technical habits are of paramount importance for the dancers: They are able to align one’s weight along the skeletal structure. They habitualize the ability to shift weight in any direction in space, independently of what the base support is (which can be any combination of extremities and/or torso), which enables reacting to subtle external impulses in any position. They remain, as a default, permeable and cultivate softness of muscle tone. Other habits are interpersonal: These include respecting constraints on dyadic geometries that allow for stable weight sharing, providing safe support in some positions, and many others. The primary purpose of good habits is to enable efficient movement, readiness, and rapport. Only if the right degrees of freedom are perfectly controlled, the body is optimally ready for situated action. However, good habits are no less creativity enabling. With an organized system of habits in place rapid motor cognition is facilitated: (1) the mind is freed for creativity; and (2) the number of effortlessly accessible affordances for action grows, notably when agents stay poised in a state of constant readiness so their body remains available for moving into any direction. 

Technical training of specific action skills is equally vital to good improvisation. Dancers learn to modulate and vary muscle tones across body parts and to use different qualities of touch to adapt in variable ways. Moreover, acrobatic skills such as rolling, falling, going upside down, falling backwards prove helpful in many situations. They learn to create “architectures” between bodies such as cantilevers and support the body weight of the partner through structural intelligence and the least amount of effort. Dancers learn to retain their agency even in disoriented states such as spinning, hanging or other upside down positions. 

Furthermore, a rich set of perceptual skills underpins improvisation. Dancers acquire kinesthetic interconnectivity, i.e., to let the senses reach forth into the partner’s body, even into distal locations, through a point of contact and send impulses there. Quite generally, our informants report that it takes particular kinds of attention to know what is going on and exploit sudden opportunities. Specific skills for “listening”—as dancers metaphorically call it—allow them to respond in the very moment. Their gaze apperceives the whole configuration. They hone their attention to tactility (friction and warmth), pressure, and their internal proprioceptive or kinesthetic states, and, when touching someone, can selectively focus on the skeleton, skin, muscles/fascia or other internal structures. Dancers learn to track their partner’s scope of attention and even recognize intentions based on incipient actions and gaze. To refine their reflexes, dancers train to become precisely aware of balance, momentum, weight distribution, weight sharing, and their partner’s as well as their own relationship to the ground. Experienced dancers are aware of the quality of touch, temperature, heartbeat, gaze, the dynamics of posture and limb placement, muscle tone, its distribution across the body, and tissue compression; they read the interpersonal body geometry, speed differentials, distances, angles, trajectories, and force vector configurations (levers, fulcrums, etc.). 

### 4.2. Skill Refinement at High Proficiency Levels

The CI teacher Nita Little ([76] p. 251) suggests that dancers should cultivate a specific kind of embodied attention that increases their proprioceptive and kinesthetic potentials:

To teach embodiment that is both located and expansive in spacetime, I support the dancers’ attentional articulation. I want to move them into the experience of the very small in oscillation with the very large (or scale changing) and places in oscillation with actions that feel spatial. This work is an extension of the attention training I learned when Steve Paxton taught the ‘small dance’ of balance as a means to study embodied conscious states. It is what he calls an ‘interior technique’ [...]. As with the small dance, interior techniques teach dancers to experience themselves as organizing spatially along trajectories and other geometries, perceiving changes and their potentials, while making multifaceted, finely tuned decisions along the way. This practice teaches a deep relaxation in counterpoint to a quickening and the ability to be touched while touching, to be changed while changing and to awaken attention to the polyrhythms of bodies, including to a body ‘standing’ in the small dance.

Similarly, our informants mention the importance of “listening” and expanding their attention, especially when entering into risky or unpredictable dynamics:

The less predictable we are, the more we have to listen to each other. […] if you have the intention of honing your attention to smaller and smaller... quantity of details. […] the thin-slicing will take you into improvisation.

With “thin-slicing”, the dancer here refers to a mode of perceptual awareness and control, which continuously stays with the ongoing dynamics. This supports motor control. It allows making a continuous stream of micro-decisions without overshooting or lagging behind, i.e., a mode of *dynamic immediacy* [77]. Dancers can now confirm or disconfirm extant dynamics with “homeopathic” responses. This means they scale their movements very precisely and just as much as they need, which a dancer describes as follows:

It’s definitely not plan making and it’s also not “when this happens, that happens” so it’s not a cause-effect learned thing. In some way, it’s even faster than thinking, in the sense of thinking as a conscious score or creating a conscious intention. It’s much faster than that. Sometimes it’s just mirroring her like responding kind of like homeopathically—like the same thing she gives me, I give back and see what comes then. Sometimes it’s kind of like feeling like completing it or following it, but sometimes it gives me an idea that is suddenly there and I just do it.

In terms of reactivity and micro-coordination, this indicates that CI dancers, instead of planning and anticipating, become capable of *intelligent reflexes* [78]. Some of our informants associate this interaction modality with a specific CI “idiom” of following the kinesthetic flow, as opposed to an extensive use of muscle force to shape and uphold acrobatic positions. The previous informant’s partner opines as follows on what makes ultra-rapid action and reaction possible in the first place:

She [her partner] said, it’s faster than call and response. So that means to me that what’s happening, has to be happening on the level of what I call, peripheral intelligence. Which is, it’s not getting up to the skull. It’s happening on the level of our interweaving as physical-mental forms. But there is creativity there, so there is some level at which choices are being made.

In this quote, the second dancer apparently attributes exceptional reaction speed to how features of the peripheral fascia and muscles are organized so that they react intelligently (for related ideas see Section 6.1).

Another special ability of experienced CI dancers is to maintain *field awareness* at each moment in the dance whereby the current situation gestures at the range of possible short-range futures. As part of pre-reflexive embodied orientations to contexts [79,80] dancers orient towards “different possibilities [that] appear on the horizon”, as one of our informants called it. CI practitioners typically notice several affordances simultaneously or even watch “alternatives attentively without using them”, as one informant dancer called it. Field awareness requires peripheral vision and, according to some dancers, non-canonical senses that expand a quasi-tactility beyond the body boundaries. This field awareness was called “spherical space” by CI founder Steve Paxton and refers to an “accumulated image gathered from several senses”. A spherical space of this kind is “the result of so many changes in spatial and kinesthetic orientation in a short time” [81]. Importantly, the concept of field awareness explains how creativity can have “partial sightedness” [82] and how dancers, accordingly, give direction to creativity, despite the irreducible role of exploration, playfulness, and chance discovery. Field awareness elucidates how dancers actively *shape the possibility space of the dance*. Dancers try “not to plan; but what I certainly do is to narrow down my options roughly”, as an informant expresses it. Field awareness provides a perceived horizon, which allows dancers to co-opt heuristics that “zoom in on a probable region of the solution space”, as creativity researcher Dietrich Haider calls it ([82] p. 901). Moreover, dancers strategically constrain this possibility space not only to make certain future more likely, but to affect how indeterminate the situation becomes as a whole (see Section 6.3). 

To sum up, our observations on perceptual acuity clearly indicate that experienced dancers: (a) track and integrate multiple sources of information; (b) know which information sources to prioritize when; (c) use this information to select or rescale their actions in real time, or anyway as fast as nerve transduction allows; and (d) know how to “read” even novel situations based on this information [40]. The account that best describes all this is ecological theory [32,35,83,84,85,86], where continuous interpersonal coordination occurs because agents reciprocally respond to momentary *affordances*. The dancers’ real-time perceptual awareness signals adaptation needs, assistance requests, springboards into familiar synergies, and perhaps a hint of a crazy creative thing to try next. One basic creativity skill is to respond quickly to ad hoc *affordances*, while another skill is to actively sculpt, tweak, redirect, or rapidly “flip” them into a new configuration. As ecological theory predicts, what dancers perceive as afforded to them or their partner is encapsulated in invariants of the movement dynamics, balance, geometry, vectors, and all the other perceptual foci that we discovered (see above). As the father of affordance theory, James Gibson, would have said, the dancers’ “educated attention” allows them to read transient informational arrays arising from the engagement. 

### 4.3. Organizing the Motor Repertoire for Flexibility

Moving from one “familiar place” to the next is not what CI is about. Dancers emphasize that they eschew using “ready-mades” and avoid chunky motor packages in general. As Nancy Stark Smith, a founder of CI, observes, “You can make a whole dance of very familiar moves. But you can also change it just a little bit to make it your own, to custom-make it in the moment—in the timing, phrasing, or the weight of it. It’s like practicing scales before you’re going to play” [87]. In fact, experienced dancers, time and again, note how they customize solutions and in doing so incorporate experiential traces from myriads of sources, which they co-assemble in real time.

To improvise effectively and come up with novel, yet functional movement material, it is imperative to organize one’s motor control system in a specific way which we term *polysynthetic*. Dancers must develop a strong capacity for variable cognitive (and motor) assembly. They must, figuratively speaking, come to freely play on the “keyboard” of CI-relevant dance parameters. In other words, dancers are faced with the challenge of assembling action in real time from a multi-dimensional array of motor sub-systems. We may analytically think of this ability as using multiple dynamic controllers pertinent to the current situation; dancers who improvise must scale and mix these controllers into fitting solutions and, even when the latter are unfamiliar, use active exploration skills and self-created feedback to “home in” on a properly balanced result. 

We may link this to the theoretical notion of *soft-assembly* [88,89]. First, this implies that agents, instead of using pre-packaged movement units, operate flexibly within a field of constraints and synthesize actions from a set of dynamic primitives. Their motor control system must be “decompositionally” organized so that (semi-)independent movement parameters can be activated on demand. Agents must scale and orchestrate these primitives in concert. This requires some awareness of how parameters influence each other (and from what point on) when the dancers re-scale them. Finely decomposable repertoires allow myriad situated assemblies and almost unbounded creativity in the dance. An infinite range of compositional outcomes can emerge from a cleverly structured competence system such as this. Soft assembly gains further purchase for explaining interaction-based creativity in virtue of the notion’s deeply ecological nature: Authors who write on the topic emphasize that soft-assembling agents exploit the coupling with external dynamics, an idea that captures well what happens in CI. Dancers attain a highly reactive fit with situated constraints and ongoing movements by complementing what the partner does through appropriately scaled dynamic primitives. They minutely adapt to parametric changes in the other body, e.g., when the partner’s muscle tone during an acrobatic movement suddenly changes, they modulate their own movement accordingly. For viable collective synergies to arise “on-the-fly”, dancers must know how to balance collective parameters, e.g., the angle and amount of weight between two leaning bodies or the way force vectors run through their body when they lift someone. A third implication of soft-assembly for creativity is that there need not be any hardwiring of modules to tasks. Parts of the motor system may be used for different things in different contexts; inversely similar outcomes can be created in different ways.

### 4.4. Coordination Skills for Unplanned, Yet Complex Patterns

Joint creativity presupposes, as a basic skill, to coordinate complex collective forms that require perfect mutual timing and complementariness. Take the case of a lift of a partner onto one’s back, and then to the shoulders to spin him there. We had dancers dissect the small-scale structure of this and other sequences in thinking-aloud to understand its complexities. It turns out that the completion of the lift demands precise co-actions over three or four stages and partners must reciprocally provide green lights—“I’m safe and ready”, as one of our informants calls it—before continuing to the next stage. Tasks such as these are multi-phasic and path dependent. The final phase is only attainable after three prior actions. 

There are many interaction researchers who argue that a keystone of complex coordination are shared task representations of “who does what when”, an implicit prior agreement (so-called *planned coordination*). Indeed, experiments on everyday actions such as joint object carrying show that short pre-agreed modules, albeit with fine-tuning potential, facilitate coordination [90,91,92]. Thus, from a naive viewpoint, this account would be tempting as an explanation of how well-timed complementary contributions in CI come about for collective forms such as lifts. The problem is that shared task representations are the very opposite of improvisation. 

Are CI patterns such as lifts a case of planned coordination, or can they provide leeway enough to deserve the name “improvisation”, and if so how? Let us look at the phenomenological data: Our experts reported that planned coordination may be used by inexperienced dancers when their real-time skills are still undeveloped. In contradistinction, at their own more advanced level, multi-phasic or otherwise complex coordination becomes possible without implicitly agreeing beforehand on “who does what when”. After all, nothing commits them to doing the full lift. They remain ready for the situation to develop in many different ways. The lift can be converted to something different at certain junctures if one partner says “no”. Our data notably provide evidence that task planning in the narrow sense rarely happens. Even in scenarios such as lifts, dancers continue to “expect the unexpected”, albeit within narrower situational constraints. Consequently, they select motor commands at the shortest possible notice, as reflexes rather than as plans. Their motor system remains ready for surprises. Whatever anticipations of possible near-futures they might have, these anticipations remain non-priming at the motor level. 

We found that the dancers’ ability to coordinate without strict planning builds on a combination of factors: First, they know they can embrace a real-time strategy since their dexterity provides enough reactivity to alleviate the need for motor anticipation. Second, once a lift begins, its biomechanics impose narrowed task constraints so skilled partners behave *relatively* predictably due to injury avoidance. Third, the situation logic itself hints at possible futures and provides a sense of field awareness, which they reported at least in lifts and other routine scenarios (see Section 4.2). This ability to “read” the situation provides a sense of the near futures the ongoing action is building up to. By perceiving the wider situated constraints (e.g., someone has both feet off the ground), dancers recognize slowly evolving aspects of the *affordance landscape* ([59], also see Section 2.2). In other words, aspects that cannot change abruptly in the next second provide crucial orientation. For example, dancers may be able to anticipate the role distribution into the near future if they are providing the support for a partner who is up in a lift. They know they will continue to be the support for about a second, irrespective of other details. Fourth, dancers understand the cumulative causal structure of sequences such as lifts and accept that “green lights” of the partner are functionally necessary at certain points to go further. Together, the last two factors—a grasp of the wider situation logic and of path dependencies—can provide the dancers with a roughly shared understanding of where they are and thus a constrained “horizon” despite indeterminacy. 

All these orienting constraints are perceived in real time. Based on them, multiple scenarios can appear on a dancer’s inner radar concerning how actions complement each other and temporally mesh with the partner’s, albeit without premature commitment to any variant. The final say belongs to the real-time triggers, while recognition of possible contingencies readies dancers. Standing prepared for multiple futures could be interpreted as *contingency sequences* hovering in both dancers’ minds, hence *possible* co-action representations (“who does what when”) that are ready to kick in rapidly, but without priming the motor system prematurely. This model explains why a multi-timescale situation awareness is sufficient to coordinate complex tasks such as lifts. Thus, instead of *prior* agreements about the task specifics, experienced dancer partners are able to create finely fitting inter-body synergies on-the-fly. Planned coordination is not needed here. The synergies are *emergently* coordinated and devoid of real planning, whether they look like a familiar “ready-made” or create a novel pattern. 

### 4.5. Creativity Techniques

To complement the picture, we now describe a set of techniques CI dancers use for creativity in the narrower sense. Creativity may take guidance from self-imposed task constraints or heuristics such as “avoid doing what comes to mind first”, “get off the beaten path/out of your own patterns”, “try to surprise your partner”, “surprise yourself”, or “try to reject offers when you can”. A similar heuristic that leads to novelty is to engage in purposeful variation and to court complexity:

There is kind of an intellectual risk or an intellectual engagement that is going on. Given it is physical and yet… I was messing constantly with how complex can I be in this moment. In how many different ways can I touch at one moment.

Improvisational creativity, at its core, involves accepting and working with whatever comes along. Dancers often emphasize how joyful “not knowing” what will happen next can be. Surprising emergence can even be courted on purpose: Uncertain, ambiguous, or even precarious zones are sought out where indeterminacy occurs, say, through a lack of physical support. Dancers take (reasonable) risks to experiment with ways of getting out of the situation. This “heightening the stakes”, as one informant calls it, jogs creativity. It challenges one’s reflexes and dexterity to rapidly find or create a resource. Dancers often mention how, for the sake of creative challenge, they relish difficult situations they could have avoided or aborted. For example, one of our participants mentioned a situation where it “takes really millisecond timing figuring out—am I being saved or am I saving?” Thus, creativity driven by external factors is invited. Similarly, a degree of provocation is held to be desirable for many dancers. Heightening the stakes in this sense depends on trusting the partner’s self-sufficiency and ability to fall safely. Another informant remarks that “joyful contact dancing is when you know, okay, the other person can look after herself, really physically and can maneuver out of various tricky situations”.

This echoes the improvisation literature where there is consensus about the importance of accepting risks [93,94]. Risk is something which improvisers use as an inspirational source for themselves or to challenge others to come up with creative solutions, as Miles Davis famously did [23,94]. For example, a recognized creativity heuristic in jazz is to immediately repair glitches by altering the wider context, where the glitch suddenly makes sense. Expressed differently: when one’s ideas fail in interaction and an instant repair or recontextualization is needed, creativity is stimulated. In one CI example that we observed, the partner failed to recognize an invitation and moved away so the first dancer ended up doing something awkward, but funny (the example will be reported in detail later and Figure 3 will show what the movement looks like). Moments such as these are not rejected, but embraced. 

Dancers mention as a creativity technique that they enter regions with indeterminate biomechanical characteristics, such as tipping points. Indeterminacy can be actively favored, or, as a dancer says: “Where is the moment that neither one of us has a clue. […] That is a place I often go to. Where is the moment that we don’t know? It is not clear for either one of us what can happen.” Where the immediate future is unpredictable, creative challenges arise. In fact, dancers may actively tip the collective system to invite (not yet determined) creative adaptations. Notably, destabilizing an ongoing coordination pattern can lead to this. In one example we observed, a dancer altered his position and initiated a rocking motion. This motion grew to the point of making him unstable and receptive to external perturbations fed into his system by the partner. In a second example, the dancers jointly adapted and oscillated between support and non-support, resulting in a constant “disassembling and assembling” of balance. The moments when she was “off being supported […] [were] really short; immediately […] [she was] clicking back in”. This example suggests that a key skill is to find new structure in the indeterminate situation. Such brinkmanship is somewhat similar to soccer players who “ride the boundary between legality and illegality to gain a competitive advantage” [95] and to martial artists who explore a tipping point to find new dynamics [64]. 

It thus appears that a technique to stimulate creativity is to seek out the analogue of what dynamic systems theory (DST) refers to as *metastable* states [95,96,97,98]. In dancing, both the individual body and the interpersonal system can occupy a *metastable* position where agents are poised at the “edge” of different possible futures, and minimal nudges can tilt the dynamics either way. We see the same phenomenon in small improvisation experiments, where experts “stay in the zone” [99,100]. To paraphrase what CI experts report, interesting possibilities result when they gravitate towards this zone full of indeterminacy, yet pregnant with multiple futures. By lingering there, they empower collective self-organization dynamics and usable fluctuations (see Section 6.2). 

### 4.6. Zoom Out

We have argued that dancers actively seek out stimulating situations, risks, challenges, and enter zones of indeterminacy. They react to self-stimulated challenges, but also stimulate the partner. They apply heuristics that constrain their own dancing to direct them away from the beaten path and to new regions of the action space. In this way, active strategies that reach slightly ahead in time are just as vital to creativity as rapid reactions to surprises and challenges. We may conclude that able improvisers actively sculpt or generate affordances that furnish creative “springboards”. 

Apart from creativity techniques proper, dancers create *conditions of possibility* for flexible and variable movement improvisation [39]. To enable creative feats, dancers need to organize their bodies and relational parameters between them for action-readiness. They stay poised for novelty. Furthermore, improvisational creativity presupposes action skills that hand the dancers a *polysynthetic*, sufficiently flexible and wide repertoire. Finally, real-time creativity presupposes “educated attention” that supports rapid decision-making even in unfamiliar situations. As dancers monitor the ever-changing biomechanical constraints, they achieve momentary orientation as well as prospective field awareness about the range of possible continuations. The latter ability prepares them for nudging things in the general direction their creativity desires. 

## 5. Results II: From Weakly to Radically Interactive Creativity

We have already introduced the notion that the ongoing engagement between the dancers, i.e., their *coregulation*, operates via a double loop of feedback and feedforward. From this reciprocal engagement, a momentary interactive milieu emerges that constrains and enables creativity, defines a field of affordances to select from, suggests springboards into new interpersonal synergies, or simply provides inspiration. We now investigate how these fundamental characteristics of the interaction relate to the dancers’ creativity. 

### 5.1. A Taxonomic Criterion

We aim to bring together a couple of concise examples to delineate the spectrum of co-creation mechanisms that CI offers to the dancers. From a synoptic perspective, the data in our study suggest that in CI we are dealing with a continuum of weaker and stronger versions of interactive co-creation. These creativity types differ regarding the role and importance of *ongoing* interactivity. We distinguish, on a relatively broad spectrum, one pole where the collective milieu simply inspires and constrains creative ideas from a pole where the ongoing coupling dynamics and co-participation between dancers serve as the *constitutive* principle of generativity.

At the weakly interactive pole, we may still speak of an “internal” mode of creativity. An idea is prefigured in one dancer’s mind to create a potential fit to the situation, and the dancer tries to implement it. The act of creation is minimally planned, and reaches into the future by perhaps a second. These internal ideas sometimes can be followed through, but more frequently they need to be adapted as the situation has already changed slightly. Even when interactive adaptations occur, the generative act lies in the prior moment and in the mind of a single dancer.

At the radically interactive pole of the spectrum, dancers let creative patterns emerge fully through the on-going process. They avoid internal ideas that would reach ahead or anticipate what they or their partner might do. Primacy is given to participatory dynamics and emergence. This means that viable, yet unplanned interpersonal synergies are created in real-time. The dancers constantly explore, respond, and, in doing so, scaffold each other over time, rather than one dancer being strongly initiating. The processually extended “in between” of two dancers is the ontologically irreducible substrate of this type of co-creation. Self-organizing couplings become constitutive. This is a distinctive feature which more internal forms of creativity lack. In fact, the forward reaching intentions of an internally creative person would actively deselect or override many fertile micro-couplings that arise in the “in between” of two dancers.

### 5.2. Minimal Entanglement and Solicited/Enforced Cooperation

In some cases, dancers report that internal creativity scarcely needs interactive adaptation to begin with. First, this happens in dancing out of contact or, in contact, when some minor flourish has little impact on the partner. Secondly, dancers can also follow their ideas freely at moments where the partner is accommodating or implicitly agrees to follow, e.g., when a dancer says “there was a moment when [he] kind of surrendered to my idea in terms of: let’s see what she is doing, and let’s accommodate that doing”. 

Another option to try to accommodate your own ideas is by manipulating the partner. For example, one of our experts with an Aikido background applied a wrist lever to force the partner into a fall (which the partner then creatively converted into a rotational figure on the ground). Besides manipulating, dancers may invite partners to share an idea of theirs, e.g., by ostentatiously extending the outstretched hand, as we observed during one of our think-alouds. Finally, dancers may create situations that offer a very limited field of affordances to the partner and will likely nudge his or her response into the desired direction.

### 5.3. Internal Creation Mechanisms

At the far end of the co-creation spectrum, an internal idea can creatively modulate familiar movement material: creatively adapting prototypes or varying the structure of known exemplars has been documented in musical improvisation [101]. In CI, working with “familiar places” such as handstands, lifts flips, or weight support in table position can proceed in a similar manner. The creative part is how the dancers tune the rhythm, spacing, dynamics, or the tactile and kinesthetic quality to the situation. A dancer commented that as soon as two people move dynamically “it is to a tremendous degree also a question of what the angles in space are precisely like […] changes in the minutiae […] have different effects in each case”. (In these cases, the creative part lies in the “how”, while the movement material itself, the “what”, can be very commonplace). Conversely, when a ready-made concerns only one body, it can combine creatively with the partner’s actions into larger patterns. 

A notch up on the creativity scale, new movement material is created. This can be as simple as using an easily done, but bizarre movement such as sticking the head underneath the partner’s shirt in order to be provocative. Creative material such as this may arise through analogies from situations such as hiding under a blanket as a child. With a dancer who is drawn to musicality, creative material can arise from a rhythm he or she pursues for a time, or a dancer may be intrigued by particular spatial variations. Since these ideas are constrained they can often only come alive fully if the partner actively picks up on the idea. In one particular case, the partner began to mimic a rhythm until dancers were rhythmically reversing roles sitting on top of each other.

Another notch up the scale, generative mechanisms from the creativity literature, can be brought into play, of which we now discuss several: The first mechanism is to recombine aspects from different familiar dance scenarios. To illustrate, we observed dancers who creatively reversed roles in the commonplace “body-surfing” scenario, where a dancer is situated on the floor and the partner in a perpendicular position on top. Usually, the lower dancer will transport the upper dancer through space by rolling across the floor. A more creatively inclined dancer in the top position, however, need not follow the momentum of the partner below. He or she can instead approach things the other way around by locking hands or feet to the floor and then moving, stopping, or redirecting the bottom dancer with his or her body center. To provide another example of how elements are creatively combined, dancers might combine a handstand with a backward movement (which is atypical and needs great skill as well as overcoming fear reflexes). In such examples, dancers mentally “mesh” known action affordances into something new (see Glenberg and Robertson [102], who noted that a shirt stuffed with leaves can serve as a pillow, if someone combines these elements after simulating their fit mentally). A somewhat related classical creativity mechanism that dancers report is grafting a familiar element to a new context. This can happen by mimicking gestures and movements or quoting other dances.

**Figure 1:**
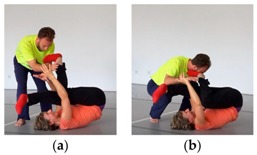
Replacing hand support with face support for partner’s legs: (**a**) hand-to-foot support; (**b**) face-to-foot support.

We may also illustrate the creativity mechanism of filling a task’s functional slots in unconventional ways: We observed a dancer (Figure 1) who replaced the canonical body part used for supporting the partner’s legs, namely the hands, to an unexpected option: his face. This solution is unusual, but fits perfectly into the dynamics since the face was already in position for giving its weight.

**Figure 2:**
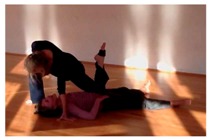
Exploring emotional bounds.

Sometimes, even full-scale analogical mappings occur. For example, a dancer said he wanted to imply a metaphorical meaning by ostentatiously falling into the partner and hereby suggest the act of “falling in love”. In another example, two dancers strode forward hand in hand and bowed like in a baroque minuet. In this way, dancers may on occasion create entire narratives in an analogical thought-space. In this particular case, only the dancer who was leading at the movement was creating this narrative, while the partner was unaware of it and focused on following without losing contact. Interestingly, however, internally created narratives originating in one dancer’s mind may be picked up on and upheld by the partner. For example, in another duet, two female dancers evoked a dominance- and eroticism-imbued story through a caress with one partner lying below the other (Figure 2). 

**Figure 3:**
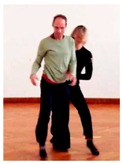
Unsuccessful lift.

To recap, internally creative CI dancers envisage a new pattern a fraction of second before trying to implement it. They do so by mentally simulating the idea to see if it is roughly functional [82], for instance by “meshing” affordances. The apparent cost of this creative strategy is that, during execution, something awkward happens because the partner’s concurrent actions are mismatched. Therefore, internally creative dancers are well advised to ensure partner compliance by implicit assistance, invitation, manipulation or nudging. Otherwise, they will probably need to adapt, redirect, or even abort their internal idea. A dancer concisely explained what the risks of fully specified movement intentions are: “The more definite imagination I put into it, the more likely I will get a no, because it will not correspond to your ideas”. In a striking example of this, a dancer experienced an awkward moment because she had over-committed herself to an idea of doing a small lift (Figure 3). She had assumed her partner was already going along with her idea, but in fact failed to communicate her invitation fully. The dancer had sensed a pre-affordance of sorts, but the lift then never got off the ground, because the all-important main affordance, the adequate support from the partner’s hips, was missing. She had interpreted the incipient dynamic as a shared preparation for her preferred co-action, while her partner felt no invitation. She remained unaware of this failure to communicate, nor did she attempt a repair. Our discussion of the event actually revealed that, *if* she had focused on the point where their bodies were to touch and had gauged the geometry, she could probably have corrected the lacking invitation on-the-fly. Instead of a late adjustment of this sort, she just stuck with her initial idea longer than was afforded by her partner’s actions. She commented that her failure had happened due to having focused too intensely on the lingering image of her desired idea.

### 5.4. Semi-Specific Ideas, Interactively Fleshed Out

Some internal CI ideas are inherently adaptable to the emergent dynamics with a partner (and thus keep the demonstrated dangers of over-commitment at bay). Many familiar dance techniques, even when a person envisages the idea to do them in advance, provide a good deal of leeway to begin with. Often, the reason is that a technique’s implementation details are not specified, e.g., in “sloughing” or creating a “rolling point” across the partner’s body. These techniques merely prescribe a *general* sort of control law, a simple behavioral algorithm that is defined relative to a collective variable, such as how a rolling point of contact moves along the partner’s body and at what angle pressure from one’s own body is directed to this point. This leaves open many paths along which to try this and any number of ways in which the limbs or torso implement the movement. In CI, the dancers’ applied knowledge of numerous familiar dance techniques revolves around broadly defined action categories, which follow an adaptable control heuristic or a general interaction principle. 

In fact, even when it is not a familiar technique dancers have in mind, they may use internally generated ideas in adaptable ways: Frequently, a person envisages mere constraints on movement rather than a fully elaborated action pattern. The dancer specifies one selected parameter but leaves most other aspects open. The dancer may in fact reckon with dynamic specification through the interaction itself to flesh the other aspects out. Cognitively, such idea material comprises what Andreas Engel calls *directives* [103], rather than plans. In our data, many directives specify a particular biomechanical function, but not its implementation. Recall the earlier example where the face touches the partner’s foot (Figure 1). Here, the intended function was to support the lying partner’s legs, and to create a stable arc between their bodies. The dancer shifted rapidly from supplying the support through his hands to supplying it with his face. Apparently, he had not committed himself to a specific motor plan in advance, but was following ensuring the arc’s architecture in whatever way possible. His partial openness regarding means allowed him to embrace a rather uncommon, but perfectly functional option that the interaction presented him with. The perhaps most frequent case of directive-like internal ideas are general dance “scores” or thematic interests that impose one particular constraint, but leave enormous leeway in all other respects, e.g., intending “to experiment with relationality without touching the partner”. A typical CI score might “fixate” a particular biomechanical parameter for 20 or 30 s, such as keeping pelvises opposite or the interpersonal distance at a particular range. This self-imposed constraint allows the dancer to flexibly exercise all other degrees of freedom. With sufficient experience, almost any external dynamics, especially anything the partner may be concurrently up to, can be accommodated without violating a creative intention of a score. (Of course, the more parameters a dancer constrains in advance the greater the challenge and risk of mismatch with the partner.) 

**Figure 4:**
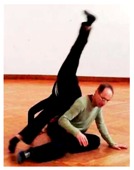
Back flipping.

Another way to remain adaptable is to creatively plan a relatively specific initial action, but to keep the later stages of execution flexible enough to incorporate partner feedback. For example, a dancer decided to flip her pelvis across a sitting partner’s bent back (Figure 4). The details only became clear after momentum had already been generated, so the dancer on top had to adapt to how the partner moved the back (in terms of directional trajectory, contact point, and amount of energy). 

**Figure 5:**
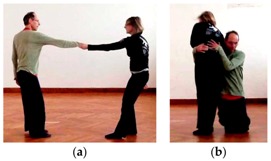
Self-experiment: (**a**) let the partner fall; and (**b**) catch them.

In a related example, a dancer has envisaged his first impulse, but left later details open. Specifically, he reported that he was trying to find means to respond to a self-created challenge to test his own dexterity by letting his partner fall backwards and then quickly trying to find a way to catch her in time (Figure 5). His adaptability was called for too, as it turned out that anything beyond a minimal version of his original plan would have endangered his partner.

To sum all this up, there is a broad category of ideation processes in CI that combine *aspectual* internal ideas with interactive specification of detail when feedback from the partner (or from one’s own actions, for that matter) arrives. This frequent creative strategy uses intentions that reach ahead in time, but eschews fully specified ready-mades and prefers something more flexible instead. Combining partial constraint settings and leaving many other degrees of freedom open to interactive tuning is inherently “improvisation-friendly” (see Section 6.4).

### 5.5. Elaborating, Echoing Motifs, and Body Memory

In a certain context, we may also speak of (partly) internal creativity when dancers pick up on aspects of the interaction dynamics to internally develop this further into new creative material. What has happened in the interaction so far may provide a creative “springboard” [104] or material to actively *elaborate*. The mechanisms of creative elaboration are recognized in different fields of esthetic creativity, including poetry [105]. In the CI context, we can apply the notion of elaboration to encompass anything from movement motifs experienced the night before down to ongoing physical impulses that inspire an idea.

In micro-scale elaboration, an internal idea is developed by moulding an active physical impulse. In one of the instances we observed, an arm-hooking action occurred with an intention that “was incidental, and then I used it more intentionally”. For instance, an impulse by the partner who has almost run its course can suggest a particular movement direction. As this category may come close to radically interactive creativity (see Section 5.6, Section 5.7 and Section 5.8), under this rubric, we restrict ourselves to cases of mere inspiration with independent execution, and leave synergistic biomechanics for later. To exemplify a merely inspirational case, a dancer “converted” a brief kicking impulse received from the partner into multiple rotations, but carried them out independently. Even if she added to the collective dynamic pattern in an esthetic sense, in a biomechanical sense, she acted more on the partner’s inspiration than to complement a physical synergy.

Much other creative elaboration happens after the inspiring physical impulse has run its course: At the temporal meso-scale, CI dancers like to speak of “compositional awareness”, a readiness to elaborate a lingering trace of perceptual memories. Dancers explain compositional dancing as involving a reflexive awareness of dance situations, for instance with respect to geometry, and a willingness to comment on, or to subvert them. Shared motifs can be repeatedly “quoted” or “echoed”, e.g., an earlier arm gesture gets mirrored in the reverse direction. Compositional dancing adds spice and dimensionality the basic operational mode of CI, as a dancer commented. It is often a momentary affordance that brings associated material and motifs back. More generally, certain imprints of moves, sensorial qualities, or themes can be, as a dancer said, “are more easily accessible to the nervous system” because they resonate with body memory [78,106,107]. Some reports from dancers would even suggest that they have a rich body-memory “sketchpad” on which they accumulate impressions. 

At the highest timescale dancers can take inspiration across longer period of interaction, again in the form of “quoting” or “echoing” accumulated imprints from the present encounter or even in the form of resonating experiences from before this dance. To exemplify the latter case, our participants report having been primed by memories of riding on a tram earlier or by memories of last night’s CI teaching session, or by recollections of repeating themes with this particular partner.

### 5.6. “Survival” Creativity

We now approach the other, more interactive end of the co-creation spectrum: Under this broad rubric some creative processes are triggered to adapt to sudden external pressures. This requires “intelligent reflexes” ([78] p. 79) that react in a flash for the sake of safety, comfort, or to avoid contact loss. This exemplifies the adaptive (i.e., not esthetically motivated) side of CI creativity and operates without prior internal design, because there is simply no time for this. 

**Figure 6:**
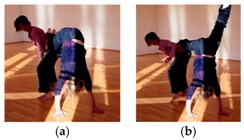
Creative adaptation emerges out of dancer’s necessity to exit a potentially dangerous situation: (**a**) the top dancer feels insecure; and (**b**) she changes into a handstand.

The creative reflex may be triggered by a glitch on either part and some unintended consequences, your partner forcing you into a tight spot or provoking you, or the simple commingling of biomechanical variables, e.g., weight vectors that tip a stable configuration into sudden imbalance. In one particular example (Figure 6), a dancer was hanging with her legs on the partner’s back and her arms on the ground (Figure 6a) and decided to kick off both legs from the back (Figure 6b), because the partner was not taking her weight fully, and she needed to move her center of gravity over her own supportive base to avoid slamming into the floor. The next moment this first adaptation was actually elaborated further by bringing her legs up fully—a handstand resulted. Even concerning the initial moment, the dancer considered the adaptive continuation creatively interesting, because her handstand emerged from a novel dynamic. 

Generally speaking, survival creativity exemplifies problem solving in response to an external challenge. Accepting the challenge provides an interesting new direction or dynamic. It supplies something to build on as well as imposing constraints for finding a solution. To use one of our informant’s words, “rather than rejecting [the challenge] or thinking you have to undo it, you emphasize it even, take it as your solution”.

### 5.7. Explorative Solution Probing, Playing, and Provoking

In addition to external adaptation pressures, a dancer’s self-produced exploratory or stimulatory activity has the power of generating creative challenges. When this happens creative *problem finding* often occurs in tandem with *problem solving*, both recognized resources [23,71]. Frequently, the two processes almost merge into one or alternate with one another. If we compare dance scenarios through this lens, we may contrast relatively open explorations with more targeted instances of so-called solution probing. Solution probing scenarios exemplify the problem-solving pole. Here the dancers feed stimulatory actions into the coupling as a way of probing for directions and useful means within defined goal constraints. Dancers, for example, subtly test the partner’s structural alignment before putting weight on him or her when they need to get out of an unfamiliar body tangle.

Other scenarios fall more to the side of problem finding, to the extent that they are more openly oriented and devoid of prior task constraints. Instead, the dancers intend to create new challenges in the first place. The many CI moments of open curiosity and playful behavior epitomize this. Playing, of course, is a well-known mechanism of creativity [38,70]: For instance, some dancers like rough horseplay. They surprise and outthink the partner by unexpected breaches of norm such as biting, slipping through the armpit, or putting the head in someone’s crotch, like “two wolves, or two cats playing with each other” and “reviving one another up” for creativity. The provocations can begin with an internal creative idea, e.g., motivated by going against expectations, but in their further course they fuel the interaction daringly. The mechanism works by interactively stimulating cascades of action and response. Since being provocative allows a broad range of responses, the dancer simply sets an interpersonal dynamic into motion, with new problems to find.

**Figure 7:**
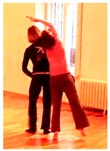
Dancers spinning in a “Tornado” theme.

A third variant positioned between these two extremes is playing with a clear (possibly shared) constraint in mind. For instance, the dancers may implicitly have agreed on a joint frame of interaction, which stimulates exploration within the frame’s open slots. In one example, two dancers spiraled around one another for several rotations, while circling around their own axes, each like a “tornado” (Figure 7). The implicit frame specified a geometric constraint: to maintain an upright vertical line and joint spatial center. Within this constraint, the dancers said that their play interest was to find out about various micro-timings and ways of intersecting, varying the rotation phasing, and dealing with the risk of colliding. Creativity might lie in the quality of interpersonal negotiation itself, regarding how the details of the joint theme are filled in. Another constrained variant is to playfully explore interpersonal and emotional boundaries, such as in the earlier example in which one dancer comes down on top of her partner, placing a knee into her soft belly parts, and gradually directing more of her own weight into her partner (see Figure 2). The dancers reported this to be “a testing on a certain kind of level of intimacy—do you trust me?”.

### 5.8. Distributed Creativity by Cross-Scaffolding

This brings us to most radically interactive pole of co-creation, where the creative process is constitutively transactional and evolves through continuous perception–action coupling: Theses processes can only be explained with reference to: (a) temporality itself; and (b) micro-cyclic cascades of reciprocal stimulation and feedback, in which the dancers’ activity stimulates further responses. We examine *micro-dynamics over time* to demonstrate how playing, solution probing, exploiting emergent possibilities, and actively sculpting the collective milieu can result in unplanned co-creation. 

We propose that cascades of micro-actions with massive parallel feedforward and feedback streams can prompt genuinely *distributed creativity* [22]. We can picture distributed creativity as indebted to the dynamics of engagement over time. This engagement provides a rich field of constraints that push towards creative solutions, an idea that has been modeled in dynamic systems approaches as “constraints induced emergence” [108]. The word “distributed” implies that a complex collective pattern emerges without originating strictly from anybody’s design. Instead, the collective pattern emerges *cumulatively* from a stream of interlacing and crisscrossing micro-actions, each of which provides a scaffold for the partner and which the latter builds on. Dancers ratchet one another like cogs on a wheel. In other words, they continuously respond to micro-affordances and embrace stepping stones provided by collective dynamics. Hence, the key to distributed creativity is reciprocal continuous enablement and inviting the partner into synergies, but also limiting, nudging, and challenging the partner—always in ways that stay with the ongoing dynamics. Dancers like to think of it as a conversation, where they say “yes, and” to small invitations, but also “no, but” or, in the extreme case, “no” flatly. Most typically, however, this cross-scaffolding happens by confirming tendencies, i.e., providing green lights for the partner, while slightly adding to the interaction in a way that makes use of existing momentum or taps into other biomechanical “freebies”. 

Analytically, we may speak of a transactional process over the time-course of a few seconds. It involves, in simultaneity, exploration and solution probing in response to the challenges that the dancers create together in real time (thus, once again, problem finding and problem solving intersect in the act). The dancers monitor the ongoing dynamics and, to quote an informant, pick up on

Nuances of the flow of movement and how weight goes through the body, through both of our bodies. […] when you work with kinesthetics, these nuances, it’s kind of like you can mold it unpredictably in a way. […] it doesn’t happen by itself. It happens by ... and also not by will. It happens by allowing it. It’s more about registering it and then micro... kind of like tiny decisions that are taken at each moment: Go for it—go against it.

Dancers often emphasize the special creative potential of this transactional stream. One informant compared these resources favorably to what we termed internal modes of creativity:

Patterns you can intentionally create and you learn […] are often relatively simple patterns and a limited repertoire. And then there are a much wider bandwidth [of patterns] which you can never intentionally produce with all their components, but which you only implicitly recognize as a chance when it arises.

**Figure 8:**
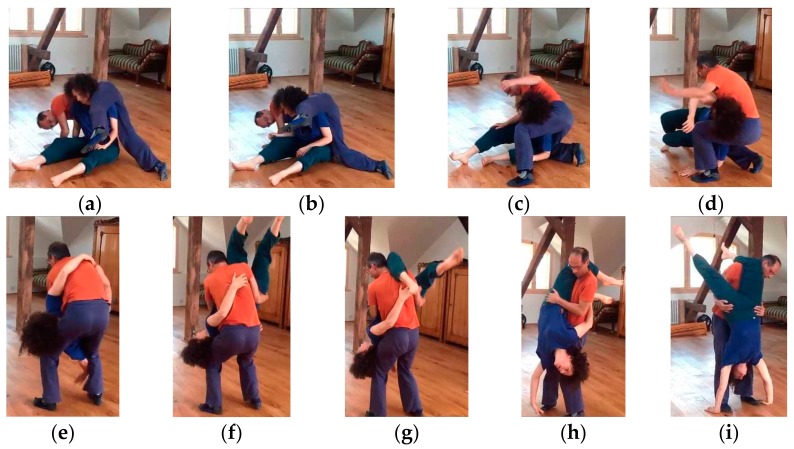
Distributed creativity through cross-scaffolding: (**a**) the male dancer hangs on their partner’s shoulder; (**b**) a transition starts—away from this position; (**c**) the dancer who previously supported weight finds a stable point pushing against their partner’s lap; (**d**) the female dancer’s pushing cumulates with their partner’s standing up; (**e**) the female dancers pulls herself up, attaching herself onto her partner’s body; (**f**) the situation culminates into an inverted lift; (**g**) the couple starts spinning as the inversion is stabilized through the female dancer’s leg; (**h**) the hanging dancer reaches out for the floor; and (**i**) and the dancer lands in a supported handstand.

The following vignette (Figure 8) illustrates the idea: It shows the entry into an inverse lift, a type of technique that dancers are familiar enough with, in general, but that started from an unusual beginning and completely without prior design. The sequence starts with one dancer sitting with her head in the lap of the other, the kneeling dancer (Figure 8a,b). As she notices the unexpected fulcrum provided by the lap (Figure 8c), she decides to exploit this micro-affordance and lift her torso upwards along his body (Figure 8d). More or less simultaneously, he decides to get up, which exerts some pull away from his partner, because he slightly sits backwards (Figure 8e). His body becomes a cantilever that synergistically adds energy to her upward motion, thanks to their strong physical connection. As the male dancer gets up more, this encourages his partner to intensify her inversion by activating the abdominals fully and to reach around him to pull herself up (Figure 8f). (She explains that, if it had not been for his sitting back, the biomechanical synergy for her continued rising would have been absent.). As he senses her rising, this provides him with a micro-affordance for adding to the dynamics further by becoming fully erect and supporting her with his arms. From this point on, a situation with familiar constraints is reached. They end up with her carried on his shoulder (Figure 8g), before she resolves this through a handstand again (Figure 8h–i). 

No one had envisaged the inverted lift; instead, both dancers responded fluently to the temporally overlapping co-actions of their partner, which supplied green lights for building on what was already there. The test criterion—and a common question in our think-alouds—is that they confirm they would have selected other options at various points without the synergistic co-actions of the partner. This exemplifies a spontaneous mutual augmentation of small dance impulses between the partners who, by continuously cross-scaffolding each other, create a viable and creatively interesting synergy. Note that, whereas the dancers were both familiar with all principles of inverse lifting, no advance planning was involved. General constraints that specify good acrobatic synergy did the job. The outcome was a physically demanding co-action without having envisaged anything. A dancer from a different think-aloud expressed the principle of how synergies emerge in this way: “it is an implicit knowledge you cannot necessarily conjure up in advance and define, instead you recognize the opportunity.” Furthermore, “I didn’t know at the time that it was a preparation. But it turned out to be useful.”

Our more general argument is that a complex interpersonal synergy can emerge from multiple couplings of smaller action components. A functionally viable cross-scaffolding process towards a (yet unknown) synergy can occur thanks to two characteristic aspects of CI: First, individual action onsets, such as the fulcrum created in the lap (Figure 8c,d), or the rising motion, are not abrupt (Figure 8e). Instead, dancers gradually scale up their actions. This allows their partners to sense nascent tendencies, to perceive hints of micro-affordances, and to co-modulate the tendencies by amplifying, continuing, supporting, buffering, or redirecting them. While one dancer picks up micro-affordances and responds with a small and continuous scaling action, this already begins to signal a complementary micro-affordance to the partner *while it happens*. Secondly, the dancers’ physical and structural interpenetration plays an important role. It ensures rapid information transmission and push-pull reactivity, but also creates a collective biomechanical unit that behaves low-dimensionally [109]. Both dancers co-regulate their joint physical unit in an effort to maintain the precisely limited degrees of freedom and force relationships that a balanced inverse lift requires (while also regulating their individual contributions to this joint action, of course).

Functionally, emergent creative patterns of this type are not easily attributable to either individuals or moments: They are causally distributed, as there is no sole author. They are also temporally distributed, as they lack sharp onsets and are multi-phasic. The phenomenological consequence of distributedness is ambiguous “ownership of the movement” [110]. Occasionally, dancers would report a, what we call, “wasn’t me” moment: they sense that neither of the dancers has initiated the movement. This phenomenon relates to a canonical CI concept: The idea of agency through a “third mind”. A dancer explicitly described the experience as follows: “there are ways and times that we aren’t separate agencies. When we come together and the fact of togetherness starts to drive what happens”. A possible explanation for this experience of agency is that dancers often go with the joint flow and momentum. It creates a strongly synergistic (and effortless) feel when an individual’s choices are continuous with the ongoing dynamics, because external biomechanics are continuously incorporated and exploited. 

We propose that dancers generate a stream of *micro-intentions* by noticing points of continuity with ongoing dynamics and by chartering it, tweaking it, or redirecting it (e.g., when they say “no, but”). Micro-intentions need not be purely reactive, though. A dancer can sculpt and at the same time cleverly exploit extant dynamics. When speaking of micro-intentions, we must not reduce these to automatic reflexes without choice or even subscribe to a collective agency that reduces dancers to puppets. They remain intentions, even though they are short-range and directed at something less complex than a full creative feat such as an inverse lift. Thus, the idea of *distributed creativity* does not imply that the individuals cease to contribute; it merely emphasizes the emerging complexity of the whole and the dynamic transactional emergence of this complex pattern. 

## 6. Discussion: Transactional Creativity from a Participatory Sense-Making and Dynamic Systems Angle

Thus far, we have demonstrated that distributed creativity phenomena can be illuminated by micro-genetic research and viewed as a process of micro-cyclic cross-scaffolding. To deepen our assessment, we now argue that distributed creativity chimes with central notions from post-cognitivist cognitive science. 

### 6.1. Co-Creation, Ecological Theory and ”4E” Cognitive Science 

Distributed creativity fundamentally owes to socio-cognitive mechanisms proposed by the enactive, embedded, embodied and extended cognition (“4E”) tradition as well as by scholars of ecological dynamics, notably in sports science. These post-cognitivist schools of thought identify contradictions in posits of internally generated agency going back to the idea of an inner homunculus that guides action; they instead highlight the importance of coupling dynamics. As ecological scholars have pointed out, the traditional view underemphasizes the role of communication and forgets about the ecological and interpersonal constraints as well as processes of dynamic interplay [111]. This new angle aims to explain how *embodied-transactional* mechanisms [112] work and, applied to our context, how these mechanisms yield creative output.

A first keystone of a transactional view is to rethink the serial “perceive-think-act” model of cognition. It needs to be replaced by the twin assumptions of massively parallel streams of perception and action and of input that can be dynamically specified while already acting [113]. Under this non-serial view, even brief actions are understood as involving multiple recursive cascades of perception–action coupling. (We have exemplified this in our preceding vignette by speaking of continuous cross-scaffolding.) Recursive micro-cycles provide the possibility to offload internal planning to repeated micro-percepts, which the agent’s active engagement makes available. Self-created feedback plays a key role in this [16,18,19], akin to when Scrabble players shift letters around to detect meaningful combinations. Thus, directed and active exploratory actions scaffold creativity. Here, no fully prefabricated solutions need to be “thought up” in advance. This *minimal cognition* copes with a task by generating stepwise solutions [114]. When the coupling dynamics over time are stimulated in a meaningful (i.e., non-random) way they can become a rich source of inspiration. Thus, micro-cyclic exploratory activity, to borrow from design theorist Schön [9], allows for *reflection in action*. 

A related, second keystone of transactional co-creation is to reject the universal necessity of fully-fledged plan representations. We have seen that in CI the co-presence of an autonomous partner makes it risky to “think up” joint action sequences in advance. Instead, dancers may well begin with sketchy ideas and flesh them out while they negotiate the course with their partner. Dancers disambiguate competing alternatives as feedback is generated. (This is also why “4E” thought emphasizes active perception [115,116] and micro-generative stimulations of the environment [117].) Both feedback and active responses from other agents can be strategically elicited. Instead of devising a fully-fledged plan, agents can follow loose *directives* that suggest further exploration foci [103], use open *task funnels* that allow dynamic specification of action details [77], engage in *constraint-based-discovery* of novel affordances [118], and in some cases even let new higher goals evolve in the process [12,13]. What is more, agents can shape or select environments to stimulate them [119,120] without pre-specifying the outcome, but by homing in on a general field of interests and possibilities.

A third keystone is the idea of *extended cognition*: Agents reach out into, and create larger functional structures with their environment. This is most evident at moments of weight sharing. A collective structure emerges that behaves lower-dimensionally in its degrees of freedom than the individual bodies. The hallmark of this *synergy* [109] lies in collective biomechanical structures such as elastic between-body architectures or levers where different bodies, or body parts from each dancer, form the two lever arms. The more general underlying idea is that an interaction dynamic can transiently create macro-systemic properties [51,121,122].

A fourth keystone is a full appreciation of the functional role that collective dynamics play and how individuals figure in the former. In essence, this depends on a coherent ontology that transcends methodological individualism, yet does not throw out the baby with the bathwater by denying individual agency. Social enactivists, who speak of *participatory sense-making* [27,28,72], emphasize that processes of interaction can partly gain autonomy when properties of the collective become temporarily self-sustaining or self-obviating. In a collective dynamic that is more than the linear sum of parts, emergent properties can add their own normativity to the intrinsic normativity of the individual agents. By further consequence, social enactivism highlights the need to skillfully regulate both individual contributions and desires as well as the coupling dynamics understood as a relational whole. (Note, however, that the emphasis on adaptive solutions in enactivism [123] and its approach to creativity [69], while being compatible with what we called “survival creativity”, shortchanges more playful, experiential, or esthetic aspects of being creative together.)

In view of this ontology, participatory sense-making theorists will likely agree with us that co-creation benefits from individual pieces of idea-material, but equally requires a regulation of a larger collective process, particularly the ability to invite more complex process outputs in ways that lie between randomness and determinism (see Section 6.3 and Section 6.4). The posit of a partial autonomy of collective dynamics sits nicely with co-creation theories such as Sawyer’s (see Section 1.2) since these also lift some emphasis away from the internalistic-mentalistic pole. In particular, the partial autonomy posit in social enactivism explains what we termed the interactive pole of the co-creation spectrum. This autonomy, however, shrinks in importance as we move towards the internal pole of co-creation.

If, in the radically interactive cases, participatory engagement becomes the very principle of co-creation, there are two alternative viewpoints on what happens here: From the individual’s angle, reciprocal engagement generates “context-dependent information fields” [124], a constant influx of new emergent affordances [125], hence inspirations and springboards into synergies to pick up on, and actions which in turn constrain or enable the partner. Meanwhile, from the third-person viewpoint the participatory micro-decisions scale up to a collective dynamic with interesting macro-properties. At this level, what meets the eye is the processes’ ability to auto-catalyze under the right circumstances [126,127,128,129]. Hence, a collective process may generate self-amplifying, self-buffering, change-resisting, delayed reaction, or otherwise non-linear properties. This auto-catalysis arises as a combined effect of “upward emergence” (micro-macro) and “downward causation” (macro-micro) [130], a dynamic which is often summed up as “self-organization“ of the interpersonal system. 

### 6.2. The DST Approach to Interpersonal Self-Organization

Before elaborating this any further, readers may be wondering about the applicability of DST concepts such as emergence, downward causation, and self-organization to our first-person viewpoint. How do notions commonly framed from a third-person viewpoint sit with skilled intentionality and creativity expertise? How do DST abstractions cash out in embodied practice? We intend to provide DST with skill-theoretical underpinnings and to deepen the heralded rapprochement between first- or second-person research and DST [131]. A qualitative angle has a key virtue—it sheds light on the micro-level foundation of the collective dynamics, where most quantitative approaches to social self-organization target collective parameters only. Moreover, our micro-genetic angle is highly fruitful for translational applications. As Alan Fogel pointed out ([112] p. 26), the informational dynamics of an interaction process can be qualitatively reconstructed and “immediately applied to the work of practitioners and participants because these models are expressed in terms of the meanings that are already present in the system”.

Much of the subjective appeal of CI lies in how dancers actively relate to a collective dynamics larger than themselves. Dancers have to (and want to) deal with the fact that the two agents’ histories, body memories, concerns, interests, current attitudes, attentional modes, and positions in space blend in unique, often non-linear ways. Any such non-linearity of process is referred to as self-organization. Self-organizing properties in a dance duet can considerably stimulate the creative process. One type of moderately self-organizing property was illustrated through path-dependent interactions, the echoing of dance motifs, or themes created through the shared dance history (see Section 5.5). The arguably most powerful self-organizing properties involve short-range biomechanical dynamics (see Section 5.8). Think, for example, of unexpected synergies between dancers when their action impulses happening at the same time amplify each other or cancel one another out, but also when subtle aspects such as floor irregularities and the position of a handstand support create fluctuations. Conversely, think of how the interplay of forces can impinge on the individual and resonate inside the body by mixing and mingling with, say, internal tensile structures or breath rhythms. The coupling patterns can fluctuate from one moment to the next thanks to a constant re-entry of feedback. When the impulses of both partners intersect “micro-blends” can result.

Technically, self-organization is a signature feature of complex adaptive systems. In principle, its effects can be seen in both single persons and interpersonal systems. Our research is inspired by a recent upsurge of scholarship on interpersonal coupling (e.g., [121,132,133]) and applications to CI [59]:

The dynamic and non-linear relationship between both and the context gives rise to specific configurations or movements and transitions among them. As in all non-linear systems the nonlinearity emerges as a consequence of self-interaction of the performers’ perceptual-motor systems. Self-interaction is manifested as a co-adaptive change between the system components: the behavior of a certain component changes itself through influencing another component, which in turn influences the first one. As a consequence of the non-linear interactions, movement configurations, i.e., patterns, arise following a process of self-organization, without the need of being consciously controlled by one of the dancers or imposed, pre-scribed, by an external agency.

Auto- or cross-catalytic processes can precipitate non-linearity such as delayed reactivity. They can also produce homeostatic behavior, as for instance reciprocal compensation of joint movers when one person is slightly “off” [109]. In dynamic systems thinking, such effects are attributed to multiple recursive couplings loops, a mix of amplifying and damping links between elements (i.e., mixed feedback). In the context of CI, candidates for couplings from which not entirely predictable collective dynamics emerge include: rhythm, momentum, speed differences in joint locomotion, the interplay of weight support and gravity or of surface friction and gravity, tipping points in cantilevers when the supporting structure slightly shifts, impulses propagating through tensegrity architectures, converging force vectors, elasticity between bodies, or autonomic mirroring of tone. We may even add interactions between attentional states to this list of couplings, which are prone to self-organization. 

In the DST literature, complex systems are, furthermore, held to display stable and self-similar macro-dynamics as long as some key setting, termed control parameter, remain the same. These systems transit to new macro-dynamic regimes when a control parameter shifts. However, this view may be too simple in the context of dance and sports science [134] where control parameters are *internal* to an individual or a collective. Collective agency is quite unlike examples where *external* parameters are manipulated to influence a system (e.g., raising the surrounding temperature to precipitate a chemical reaction in a fluid). In CI dancing, the level of dance skills and the participants’ attention, interests and preferences, as well as intentionally selected dance “scores” comprise internal control parameters, while external control parameters most likely relate to the available amount of space and time. 

Dancers frequently emphasize how kinesthetic interconnectivity itself unlocks non-linear creative resources. A duet creates a constantly shape-shifting collective unit when one dancer actively reaches forth into the body structure of the other. A great potential for parallel micro-couplings (i.e., mixed feedback) accrues from weight sharing, which allows different physiques, skills, and moods to blend in multi-faceted ways. There are constant shifts within how body parts or sub-movement dynamics loop together: (a) regarding the degree of behavioral coupling; (b) regarding its physical location; (c) regarding its experiential and sensory modalities (surface touch, tissue compression, heat, gaze, etc.); and (d) regarding the very coupling principles (merely informational coupling vs. physical push-pull). Especially, “thin-sliced” sensorial discrimination and rapid reactivity can heighten the collective self-organization potential. Thanks to these special skills, dancers begin to tweak, augment, transform, or thwart their partner’s impulses even as they are arising, i.e., they *co-modulate* each other’s actions in real time [41]. We believe that co-modulations are highly prone to stimulating interesting dynamics down the line.

Many practitioners report interpersonal self-organization of a radical sort, saying things such as “the meeting itself creating the dance as opposed to one person proposing and the other person responding” or “togetherness starts to drive what happens”. Sometimes experiences of unexpected ease, even “interaction magic”, result. These first-person observations, we argue, can be linked to the third-person notion of *interaction-dominant dynamics* [135], where the opposite are component-dominant dynamics (in which collectives are linear sums of the parts). In interaction-dominance, interpersonal autocatalysis becomes more powerful than the intrinsic dynamics of individuals. Of course, interaction-dominance does not usually arise by biomechanical automatism. It may occur when CI dancers accord interaction this power. They must put aside “solo” intentions, seek coupled behaviors, be open to subtle input, and calibrate their bodies for biomechanical “freebies”. Interaction dominant properties are thus most manifest in cases of “distributed creativity”, which capitalize on the dancers’ willingness and ability to pick up on micro-scaffolds of the partner and environment in real time. 

### 6.3. Emergence Management Skills

The importance of agent intentions and skills has powerful implications for the theory of skilled intentionality in collectives. Even in distributed creativity when collective patterns cannot be fully controlled, much of the time these do not just “happen to you” either. Instead, agents skillfully relate to collective emergence and give it some direction. To explain this strategic way of shaping a collective process, we need to discuss a suite of skills dedicated to monitoring, anticipating, constraining, impelling, exploiting, and otherwise tweaking emergence. Hereby, experts both embrace self-organization in progress and relate to it *reflexively* in informed and strategic ways.

We claim that CI experts—similar to any expert dealing with a complex system—recognize what the system is roughly responsive to at a given moment [136]. For instance, able dancers can anticipate which situations are prone to high degrees of interaction-dominant dynamics, as defined above. Dancers report that this largely depends on momentum, on whether the action is ballistic, and on how pro-active the partner’s current endeavours are. As our discussion of metastable states indicated, dancers also exercise considerable control over how indeterminate the collective situation (and thus how wide the affordance field) becomes. Furthermore, dancers report that they can often extrapolate from ongoing dynamics when slight tweaking suffices and when a real impulse is needed to get what one wants. 

At a macroscopic scale, dancers may tweak self-organization by calibrating *control parameters*, i.e., cognitive or biomechanical parameters that are powerful enough to non-deterministically influence many other parameters in the system. By tuning and setting a well-selected control parameter, a dancer might invite micro-couplings of a particular type. For instance, one study participant mentioned that the mixture of individual dance “scores” (which were mutually unknown) led to highly dynamic dancing over several minutes, and generated physical rebounds as an interesting resource for creativity. Their individual cognitive control parameter settings were quite different here, but in sum both dancers welcomed the creative potential of the resulting dynamicity in their dancing and so decided to stick to their initial score. 

At a mesoscopic scale, dancers recognize system configurations in which minor biomechanical facets can suddenly provide massive leverage and flip the collective biomechanical situation into something different. Experienced practitioners strategically manipulate certain properties, knowing this will likely stimulate new potentials (cf. [41]). To exemplify, getting up from the ground into a leaning position predictably opens up degrees of interpersonal freedom. Conversely, extensive physical contact that includes most of the body surface of both dancers can at times restrict options to move. A dancer might decide to retain the weight sharing dynamics, but employ the extremities and keep the partner at “arm’s length”, to make a broader range of micro-dynamic options likely.

At the micro-scale, dancers harness incipient biomechanical tendencies to their needs. The simplest case was already discussed—the exploitation of momentum-based or gravity-based continuity in the joint biomechanical system. Rather than working against dynamics, dancers simply jump the bandwagon to make use of something readily available. Doing this, however, is a deliberate decision, as expressed by one of the experts in our study: “I had a choice of not doing it or doing it. I could have stayed with it but it was like—OK, I want to change”. In other cases, dancers actively exploit collective momentum, bounciness, *tensegrity* of the fascial systems, bone alignment in plumb lines or in angles, elasticity of body tissues, or bounciness of the dancefloor. They can cleverly deflect an impinging force. They can also use push-pull features such as giving a small impulse and the shaping the reverberation. 

At the most imperceptible level, perhaps, it is possible to skillfully exploit *intrinsic dynamics* of the body. Even in relatively static positions movement can be created from micro-movements of breathing, muscle twitches, and “small dance” of the body, as the ongoing micro-negotiation of upright balance is called in CI. Dancers report that the gentle bobbing to-and-fro or breathing can be used to initiate larger movements. Almost imperceptible potentials can thus be augmented for interesting effects to arise. Experts also “charter” collective physical tendencies through small, but strategic enabling actions. Consider the example of tone reduction, which a dancer strategically used to follow an existing physical wave:

[…] your weight comes to the one side but then as it reverses it creates a wave and I just follow it”. […] Following the wave also requires proficiency because you have to relax certain parts of your body in order to follow. […] Reducing the tone is also a very active thing to do.

This quote is noteworthy in that it directly mentions the skills it takes and how the dancer exploits micro-trends so that internal and external agencies coalesce into an emergent effect. 

### 6.4. Joint-Improvisation-Friendly Intentions

We have so far argued that collective self-organization trends are constantly infused with skilled intentionality; even highly interaction-dominant processes are given direction in informed ways. One essential aspect—ever-present in the conversations of CI dancers—is still missing from this argument. It concerns the question of general intentional constraints each dancer brings to an otherwise open encounter.

Let us briefly take a step back again: It is true, as reported in Section 5.2 and Section 5.3, that dominantly internal creativity can occur whereby a dancer envisages a *full movement intention* that very closely constrains motor activations for one’s own body before the act. However, CI dancers—much of the time—tend to eschew fully specifying plans that reach ahead. The risks are too evident, as expressed in an earlier quote: “The more definite imagination I put into it, the more likely I will get a no, because it will not correspond to your ideas”. Keeping intentions tentative or schematic until the moment arrives is more functional. Motor commands are created in the last moment to fit the ongoing interaction dynamic, as expressed here: “In an improvisation situation, it tends to be the case that I, as it were, narrow the frame to a certain selection of alternatives, and in the moment where we, for example, meet in contact it becomes this one option.” 

If intentions are rarely envisaged as fully specifying plans, what do they consist of the rest of the time? Some parts of the answer were already given earlier: First, dancers report *aspectual full intentions* that reach ahead a little, yet leave other biomechanical parameters unspecified (e.g., intending to keep the hips in a specific geometry, but readily varying all other parameters). Second, dancers constantly deploy *micro-intentions* that are short-lived and mostly say “yes, and” or “no, but” to ongoing dynamics. These micro-intentions are precisely in sync with the dynamic and never reach ahead more than would endanger this fit. 

Third, dancers impose *non-deterministic* intentional constraints at a higher timescale, which we began to discuss under the rubric of control parameters in the last section. Such higher-timescale constraints and directives come in many types: The most frequent reported type are “scores” that define general aims without any specified implementation, thus a *global intention* such as “remaining close to her but not in physical touch”, “feel into the bone structure of my partner through the tactile interface”, “explore the vertical dimension”, “explore rotations around one another”, or “move as little as possible”. Next, dancers report *thematic interests* to pick up on when the opportunity arises and *task type intentions*, e.g., general, but otherwise unspecified interests in rolling point techniques, handstands, lifts, “sloughing”, ground techniques, etc. 

We may sum these types of non-deterministic intentionality up as *framing intentions*. Framing intentions constrain what from the interactive process to pick up on, yet remain too schematic to narrow down options to a specific realization. This makes them compatible with multiple realizations and allows exploiting a wide range of emerging micro-affordances. In other words, framing intentions remain amenable to dynamic specification. Framing intentions are inherently “joint-improvisation-friendly”; they do not clash with interactive emergence, but supply mere constraints on it. 

Dancers, and we as theorists alike, conceive of interaction-based intentionality quite differently than philosophers or psychologists usually do. Much CI intentionality is high-level and schematic in content and nowhere near fully specifying action plans. CI accentuates an important insight of DST scholarship here: No kind of intention can *strictly* cause dynamically adapted co-action behavior all by itself. It can at best furnish “constraints on dynamics” [137] within the coupling context [84,121,133,135,138,139]. The idea of constraints on dynamics converges in striking ways with the subjective reality of dancers who respect collectively emergent processes. This attitude is most compatible with, as one dancer said, “a general hunger for” and fleshing out the specific moves happens in real time by “listening to the dynamics”. This quotation rather programmatically expresses that CI agents mostly prefer to constrain their intentionality globally, and wait for the moment to decide.

Within this process, the role of framing intentions is to generate directed activities that co-determine a general direction of the duet. At the level of cognition, framing intentions impose thematic decision filters that prioritize some of the many arising micro-affordances. They also guide active exploration and strategic feedback-generation in keeping with the notion of loose *directives* [103]. A dancer nudges the dynamics until an affordance arises that fits the bill. Only the real-time perceptual feedback concretizes the action:

So my intention was to find a way to get in touch with him again and I didn’t know exactly where I would meet him and which part of his body and which part of my body would really touch. And then from this moment when I was leaning and getting in touch with him… there it was kind of like “oh, this could open up into an arch” to keep in touch with him. So this decision came through the touch. The moment of touching showed me the potential of where to go to. Through the moment of touch, I decided to keep further in touch with him. I need to open my chest because he moves back and this opening the chest brought me to this “ah! Let’s keep opening further”. […]

It is thus ultimately the rapidly evolving collective biomechanical milieu that narrows down how individuals contextually concretize framing intentions. Usually, the mix of two dancer’s currently active framing intentions drive the collective to some region of the interaction space where their further engagement narrows down possible micro-realizations or lets new micro-ideas emerge. 

To recap all this, we have proposed an *intentionality hierarchy* across timescales where micro-intentions take on shape situationally under constraints provided by higher-timescale intentions such as scores, themes or preferred task types. Micro-intentions emerge more or less at the timescale of motor commands themselves. Our present emphasis thus stands in contrast with hierarchical models of action [140] where lower-level intentions are guided by rather well-defined higher-level ones. Instead, CI thrives on nested levels of intention where the higher levels are schematic, hence highly non-deterministic, and the lower levels only develop when the moment arrives. All levels, including weakly specifying general constraints, are a genuine part of *skilled intentionality* towards the interaction [80]. This dynamics sensitive model of creative intentions nicely explains how dancers achieve a situated balance between openness to surprise and leaving a personal imprint. The presence of constraints makes co-creation all but arbitrary. Recall the empirical studies reported in Section 2.2, which indicate that the breadth of exploration benefits from a *useful* degree of constraint and adaptive pressure (whether self- or other-imposed). 

An ecological model of co-creation would then ask which variables guide the agents in narrowing down the solution space, whereby their momentary micro-intentions can take on shape. This is a process of convergence, as we have seen. Prior intentional constraints—to varying degrees and at different timescales—blend into the emerging spatial and interactional constraints. In this complex equation, the perceived available space, collective milieu, readiness, and skill define the field of affordances available to a dancer. In CI, at least in most situations, dancers may freely select among these affordances. However, they are guided by their own framing intentions, their moods, habits, and implicit preferences (e.g., concerning risk, acrobatics, experimentation, or mode of rapport), as well as by collective properties of the duet, be they physical (e.g., in terms of size and weight differences), related to histories of dancing together, or of other sorts. 

Having come full circle now, we must reiterate that intentions and collective dynamics can be integrated in different ways. Dancers exercise variable ranges of “projection span” into the future and may or may not insist on prior intentions. In what we called *weakly interactive co-creation* a dancer tries to implement an idea which fits the situation at the outset, but has a projection span of a second or more, and may not fit the subsequent dynamic unless one ensures partner assistance, enforces the idea, or is only engaged in weak physical coupling with the partner at this moment. In contrast, in *distributed creativity*, unintended forms arise through a micro-cyclic process of continuous cross-scaffolding. It is thus a pertinent question for scholarship to inquire into the different possible modes of balancing constraining intentions and the willingness (and ability) to embrace surprise, serendipity, and emergent structures. 

## 7. Conclusions

Our objective was to survey creative modes and resources in the dance domain CI to provide, as Keith Sawyer posited it over a decade ago, an *interactional semiotics* of co-creation, extended by the factor of embodiment and joint kinesthesia. We described how creative (i.e., novel and functional) dynamics in a duet emerge and we provided a vocabulary for this process. 

The main novelty value of this paper lies in contrasting forms of performative co-creation on a gamut from relatively “internal” to dominantly “interactive” forms of ideation. We can sum up the argument in four points: (1) In co-improvised embodied performance, the generation of movement ideas is both constrained and inspired by a stream of participatory engagement. (2) Although creativity is always interactive, it ranges from weakly to strongly participatory versions—we have distinguished internally generated, but interactively implemented ideas and fully participatory (i.e., distributed) types of creativity that operate by continuous cross-scaffolding. (3) CI experts mostly prefer forms of skilled intentionality that are of a “joint improvisation-friendly” kind, which balance collective constraints with individual creative freedom. Emergence is respected by guiding, tweaking, and nudging an ongoing collective dynamic, but also by allowing things to unfold. (4) Methodologically, we demonstrated how to put into practice a thorough micro-analysis of transactional emergence, which tracks precisely how intentionality weaves itself into the process. This shows how co-creation research can go well beyond topics such as brainstorming and explore the implications of real-time dynamic coupling as a generative principle in its own right.

As a second innovation, our survey of creativity related skills made efforts to distinguish between two levels. First, dancers train a range of sensorimotor skills to enable improvisational co-creation, i.e., to supply necessary *conditions of possibility*. This includes skills for remaining poised in readiness; a functional attentional focus on “what matters”; perceptual “thin-slicing” and real-time reactivity; motor repertoires that are capable of combinatorial synthesis; and capacities for micro-coordinating complex movements without planning ahead. Second, creativity techniques in the narrower sense were analyzed, notably heuristics for seeking novelty, for self-stimulation, and for gravitating towards micro-zones of the action space where novelty is likely to arise. We also connected the idea of *distributed creativity*, the CI modality where creativity arguably becomes richest, to abilities for real-time cross-scaffolding with the partner. Distributed modes of creativity put high demands on all of the above enabling skills, but also require a knack for inviting and exploiting self-organizing dynamics. This surplus hardly “just happens”—it requires ongoing “emergence management”, a set of special skills that allow reaping the benefits of embodied or interpersonal auto- or cross-catalysis. Overall, the present case study suggests an important new focus for dynamic systems research, namely the study of expert skills for properly calibrating, nudging, tweaking, and optimally exploiting a complex non-deterministic system.
